# Measurement of person-centred consultation skills among healthcare practitioners: a systematic review of reviews of validation studies

**DOI:** 10.1186/s12909-023-04184-6

**Published:** 2023-04-05

**Authors:** Anne van Dongen, Duncan Stewart, Jack Garry, Jim McCambridge

**Affiliations:** 1 Department of Psychology, Health, and Technology, University of Twente, Enschede, the Netherlands; 2 School of Social Sciences and Professions, London Metropolitan University, London, UK; 3 Department of Health Sciences, University of York, York, UK

**Keywords:** Person-centred, Patient-centred, Measurement, Consultation skills, Healthcare practitioners

## Abstract

**Background:**

Person-centred care is integral to high-quality health service provision, though concepts vary and the literature is complex. Validated instruments that measure person-centred practitioner skills, and behaviours within consultations, are needed for many reasons, including in training programmes. We aimed to provide a high-level synthesis of what was expected to be a large and diverse literature through a systematic review of existing reviews of validation studies a of instruments that measure person-centred practitioner skills and behaviours in consultations. The objectives were to undertake a critical appraisal of these reviews, and to summarise the available validated instruments and the evidence underpinning them.

**Methods:**

A systematic search of Medline, EMBASE, PsycINFO and CINAHL was conducted in September 2020. Systematic reviews of validation studies of instruments measuring individual practitioner person-centred consultation skills or behaviours which report measurement properties were included. Review quality was assessed with the Joanna Briggs Institute Critical Appraisal Checklist for Systematic Reviews and Research Syntheses. Details of the reviews, the included validation studies, and the instruments themselves are tabulated, including psychometric data, and a narrative overview of the reviews is provided.

**Results:**

Four reviews were eligible for inclusion. These used different conceptualisations of person-centredness and targeted distinct, sometimes mutually exclusive, practitioners and settings. The four reviews included 68 unique validation studies examining 42 instruments, but with very few overlaps. The critical appraisal shows there is a need for improvements in the design of reviews in this area. The instruments included within these reviews have not been subject to extensive validation study.

**Discussion:**

There are many instruments available which measure person-centred skills in healthcare practitioners and this study offers a guide to what is available to researchers and research users. The most relevant and promising instruments that have already been developed, or items within them, should be further studied rigorously. Validation study of existing material is needed, not the development of new measures.

**Supplementary Information:**

The online version contains supplementary material available at 10.1186/s12909-023-04184-6.

## Background

Person-centred care (also termed patient-centred care [1]) has been widely acknowledged as an essential element of high-quality health service provision [2]. The concept of person-centredness has been utilized for roughly half a century and has been applied at different levels, from national healthcare policy to skills as specific as non-verbal communication behaviours [3]. Many different perspectives on, and definitions of, person-centredness exist, thus making it a somewhat contested concept to operationalise [1, 4]. Arguably, these are variations in emphasis within a core theme, though they do have implications for valid measurement.

Consultations are a key component in health care provision which offer an opportunity for patients to discuss issues with practitioners. Practitioners often have multiple tasks within consultations, including eliciting information to aid assessment, and information-giving. Individual practitioners vary in consultation skills and commitment to make the conversation person-centred in practice [5, 6]. In the past two decades person-centred communication skills acquisition has received much greater attention in training programmes [7, 8]. To evaluate the efficacy of training programmes designed to enhance person-centred skills, validated instruments that objectively measure these skills and their use in practice are needed.

Systematic reviews of validation studies of instruments measuring person-centeredness were known to exist prior to undertaking this study, however, it was clear that this literature was diverse, and that such reviews may have different purposes, aims, and inclusion criteria. Reviews have been aimed at identifying and/or appraising instruments for specific conditions (e.g., cancer, [9]), health care settings (e.g., neonatal intensive care units, [10]), or professions (e.g., psychiatrists, [11]). In addition, across existing reviews different conceptualisations of person-centredness frame research questions and selection criteria in distinct ways (e.g., see [12–16]). Consequently, there may be little overlap in the primary studies included in available reviews, and no one review summarises and evaluates the literature as a whole. For these reasons we aimed to provide a high-level synthesis of this complex literature by undertaking a systematic review of reviews. This was intended to provide an overview of how existing systematic reviews are designed and report on validation studies, and to incorporate details of the included instruments. This study thus brings together what is known about available instruments that may be considered for use in training and assessment of person-centred consultation skills among healthcare practitioners, for researchers and research users. This review of reviews was thus not undertaken to identify a particular instrument for a particular purpose, but rather to survey the level of development of, and the strength of the evidence available in, this field of study.

Reflecting these aims, the objectives of this review of reviews were to: 1) undertake a critical appraisal of systematic reviews reporting validation studies of instruments aiming to measure person-centred consultation skills among healthcare practitioners, and 2) identify and summarise the range of validated instruments available for measuring person-centred consultation skills in practitioners, including material on the strength of the validation evidence for each instrument.

## Methods

This review followed the process outlined in this section, which followed the development of a study protocol prior to the conduct of the review. We did not prospectively register or otherwise publish the protocol.

### Search strategy

Systematic searches were conducted in the electronic databases MEDLINE, EMBASE, PsycINFO, and CINAHL. The search strategy combined different search terms for three key search components: ‘person- or patient centredness’ (Block 1), ‘assessment instrument’ (Block 2), and ‘systematic or scoping review’ (Block 3).

For Block 1 (the search component ‘person- or patient centredness’) we used an iterative approach. A preliminary search of EMBASE, MEDLINE, and PsychInfo (all in Ovid) was undertaken using the keywords: (person-cent* or patient-cent* or personcent* or patientcent*) and ‘review’ in the title; and ‘measurement or tool or scale or instrument’; from 2010. Full text papers identified (*n* = 24) were searched for words used to describe ‘person- or patient centredness’. The resulting search terms were discussed and selected to reflect the scope of the study. The final search included the following terms: person-cent* or patient-cent* or personcent* or patientcent* or person-orient* or person-focus* or person-participation or person-empowerment or person-involvement or patient-orient* or patient-focus* or patient-participation or patient-empowerment or patient-involvement or "person orient*" or "person focus*" or "person participation" or "person empowerment" or "person involvement" or "patient orient*" or "patient focus*" or "patient participation" or "patient empowerment" or "patient involvement"; or (clinician-patient or physician–patient or professional-patient or provider-patient or practitioner-patient or pharmacist-patient or doctor-patient or nurse-patient) adjacent to (communication* or consultation* or practice* or relation* or interaction* or rapport).

For Block 2 (the search component ‘assessment instrument’) we used the existing COSMIN filters proposed by Terwee et al. [17]. The COSMIN (COnsensus-based Standards for the selection of health Measurement Instruments) project has developed highly sensitive search filters for finding studies on measurement properties [17]. The search filter was adapted to each database. For Block 3, the search terms (systematic* or scoping) adjacent to review* were used. The search did not include restrictions pertaining to date of publication, and the language was restricted to English. The database search was conducted in September 2020. See appendix 1 for the details of all searches run in all databases.

### Study selection

One author (JG) screened titles and abstracts against preliminary selection criteria, using Rayyan software for systematic reviews [18]. Ideally all parts of the process of undertaking a review are duplicated to in order to avoid errors. Here we relied on one author for screening, with the rationale was that we expected systematic reviews to be readily identifiable in the title and abstract, making screening more straightforward, for example, than in conducting a systematic review of primary studies, which may be described in more heterogeneous ways. Another author (AD) screened 5% independently. The authors met weekly to resolve any problems or questions during the process and no contentious issues were identified in screening. Full text articles of potentially eligible papers were retrieved and assessed for inclusion against the criteria below. Two authors (AD & JM) reviewed all full text papers independently in order to select studies for inclusion. One disagreement was resolved through discussion with a third author (DS) and reasons for exclusion were noted. Inclusion criteria were:

- a peer-reviewed journal report
- used systematic review methods to identify primary studies for inclusion (including both a search strategy and explicit selection criteria)
- stated aims and objectives specifying the measurement of ‘person centredness’ or ‘patient centredness’ or a related construct as defined by search Block 1.
- concerned assessment of individual practitioner consultation skills or behaviour (i.e., not policy)
- included only validation studies of instruments
- reported any measurement properties of the included instruments

**Fig. 1:**
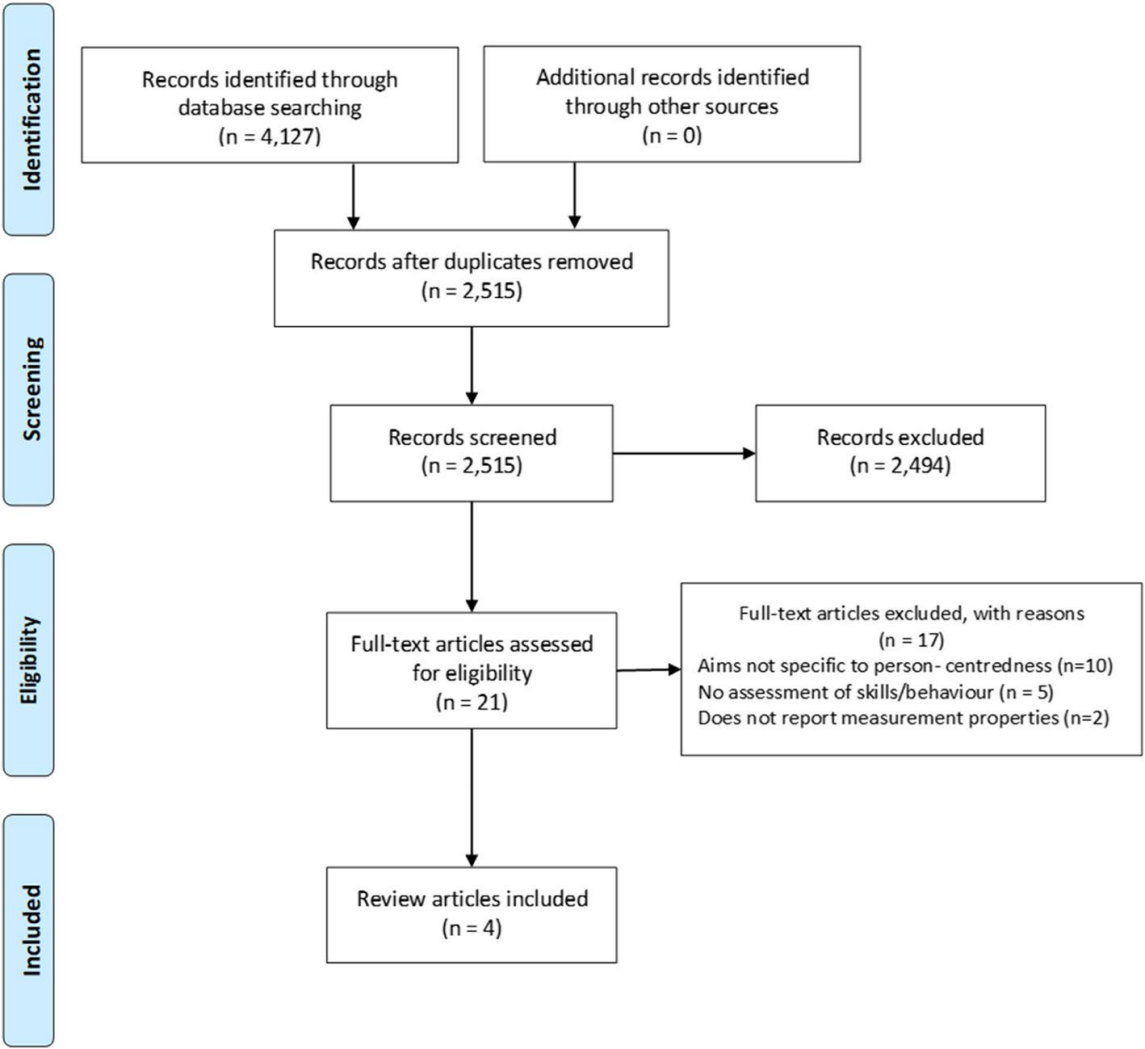
PRISMA flow diagram

Reviews of instruments developed for any practitioner group, patient population, or health care setting were included. Studies were excluded unless they met all inclusion criteria. After the full text eligibility check, a backwards search of the references of the included reviews, as well as a forward reference search using Google Scholar was performed. This was last updated in January 2022 and no further reviews were identified. A PRISMA flowchart [19] shows the results of the identification, screening, and eligibility assessment process (Fig. 1).

### Data extraction

One author (AD) performed data extraction from the included reviews using a standardised form created in Excel developed by all co-authors in a preliminary phase. A second author (DS) subsequently checked all the extracted information in the form, and screened the paper for any missing information. At the review level, we extracted the stated aims and objectives, definition or conceptualisation of person-centredness used, numbers, names and types of instruments, research questions, dates, databases, and languages included in search strategies, selection criteria regarding health care populations, health care settings, raters of the instruments, other selection criteria, details of the assessment of methodological quality and psychometric properties, and numbers of validation studies. At the validation study level, we extracted the country of origin, the type of validation study, and whether the developers of the instrument validated their own instrument. At the instrument level we extracted who developed the instrument, in what year, in which country and in what language the instrument, how many subscales and items the instruments consisted of, and the response formats used. Other information on validation studies and instruments was not reported consistently enough to be extracted.

### Quality assessment

Two authors (AD & DS) independently assessed the quality of the included reviews using the Joanna Briggs Institute Critical Appraisal Checklist for Systematic Reviews and Research Syntheses checklist [20]. Each of the 11 criteria was given a rating of ‘yes’ (definitely done), ‘no’ (definitely not done), ‘unclear’ (unclear if completed) or ‘not applicable’. Discrepancies in the ratings of the methodological reviews were be resolved by consensus.

## Results

### Description of the reviews

The search identified 2,215 unique articles with 21 papers selected for a full-text eligibility assessment (see Fig. 1). Four studies were included. None of the reviews identified in further searching fulfilled our inclusion criteria.

The four included reviews each had different aims and selection criteria, resulting in few primary studies and instruments being included in more than one review. Two reviews targeted different groups of practitioners; nurses for Köberich and Farin [21] and physicians or medical students for Brouwers et al. [22]). Hudon et al. [23] and Köberich and Farin included only patient rated instruments, while Ekman et al. [24] included only direct observation tools (e.g., checklists or rating scales). In total, the four reviews included 71 validation studies (68 unique studies) of 42 different instruments.

### Conceptualisations of person-centredness

Conceptualisations of person-centredness varied between the included studies. Two reviews used Stewart and colleagues [15] model of interconnecting dimensions: 1) exploring both the disease and the illness experience; 2) understanding the whole person; 3) finding common ground between the physician and patient; 4) incorporating prevention and health promotion; 5) enhancing the doctor–patient relationship, and 6) ‘being realistic’ about personal limitations and issues such as the availability of time and resources. Dimensions 4 and 6 were later dropped [14]. Brouwers et al. [22] included instruments measuring at least three out of the six dimensions, while Hudon et al. [23] included those measuring at least two out of the later version of four dimensions. Köberich and Farin [21] used a framework of three core themes of person centredness based on Kitson et al. [13]: 1) participation and involvement; 2) relationship between the patient and the health professional; and 3) the context where care is delivered. Finally, Ekman et al. used an Institute of Medicine framework [16] of six dimensions: 1) respect for patients’ values, preferences, and expressed needs; 2) coordination and integration of care; 3) information, communication, and education; 4) physical comfort; 5) emotional support, e.g., relieving fear and anxiety; and 6) involvement of family and friends (Table 1).

**Table 1:** Overview of reviews

Study	Stated aim	Definition or conceptualisation of person-centredness	Population	Setting	Rater	Other selection criteria	Assessment tool used	N of studies included	N of instruments
Hudon et al., 2011	Identify and compare instruments, subscales, or items	The four dimensions common to Mead and Bower’s review and Stewart et al	Not specified	Ambulatory family medicine	Patient	Measuring at least two dimensions	Modified Version of STARD	26	13
Köberich & Farin, 2015	Provide an overview of instruments	Kitson et al.’s three core themes	Nurses	Not specified	Patient (adults only)	Measuring at least two of the core themes	None	12	4
Brouwers et al., 2017	Review existing instruments	Stewart et al.’s six dimensions	Physicians or medical students	Not specified	Not specified	Measuring at least three dimensions	COSMIN	13	14
Ekman et al., 2020	Review and evaluate direct observation tools	The framework endorsed by the Institute of Medicine	Not specified	Not specified	Direct observation	Excluding clinical encounters	None	19	16

### Overview of reviews

Hudon et al.’s review [23] aimed to identify and compare instruments, subscales, or items assessing patients’ perceptions of patient-centred care used in an ambulatory family medicine setting. Only patient rated instruments were included. Quality assessment of the validation studies was conducted with the Modified Version of Standards for Reporting of Diagnostic Accuracy (STARD) tool [25]. The authors identified two instruments fully dedicated to patient-centred care, and 11 further instruments with subscales or items measuring person-centred care.

Köberich and Farin’s review [21] aimed to provide an overview of instruments measuring patients’ perception of patient-centred nursing care, defined as the degree to which the patient’s wishes, needs and preferences are taken into account by nurses when the patient requires professional nursing care. Again, only patient rated instruments were included. The four included instruments were described in detail, including their theoretical background, development processes including consecutive versions and translations, and validity and reliability testing. No quality assessment was undertaken.

Brouwers et al. [22] aimed to review all available instruments measuring patient centredness in doctor–patient communication, in the classroom and workplace, for the purposes of providing direct feedback. Instruments for use in health care professionals other than physicians or medical students were thus excluded. The authors used the COSMIN checklist for quality assessment of the instruments [26].

Ekman et al.’s review [24] aimed to identify available instruments for direct observation in assessment of competence in person-centred care. The study then assessed them with respect to underlying theoretical or conceptual frameworks, coverage of recognized components of person-centred care, types of behavioural indicators, psychometric performance, and format (i.e., checklist, rating scale, coding system). The review used the six-dimension framework endorsed by the Institute of Medicine [16] however, they did not use the framework as a selection criterion. No quality assessment was undertaken. The authors group the included instruments in four categories: global person-centred care/person centredness, shared decision-making, person-centred communication, and nonverbal person-centred communication.

The critical appraisal of the included reviews using the Joanna Briggs Institute Critical Appraisal Checklist for Systematic Reviews and Research Syntheses is reported in Table 2. The review by Brouwers et al. [22] scored positively on all but one items. We note that no study assessed publication bias, and this may be a particularly important threat to valid inference in a literature of this nature. There were issues with the methods of critical appraisal in two reviews.

**Table 2:** Critical Appraisal

	Hudon 2011	Köberich 2015	Brouwers 2017	Ekman 2020
Is the review question clearly and explicitly stated?	✓	✓	✓	✓
Were the inclusion criteria appropriate for the review question?	✓	✓	✓	✓
Was the search strategy appropriate?	✓	✓	✓	-
Were the sources and resources used to search for studies adequate?	✓	-	✓	✓
Were the criteria for appraising studies appropriate?	✓	-	✓	-
Was critical appraisal conducted by two or more reviewers independently?	✓	-	✓	-
Were there methods to minimize errors in data extraction?	✓	-	✓	-
Were the methods used to combine studies appropriate?	-	✓	✓	✓
Was the likelihood of publication bias assessed?^a^	-	-	-	-
Were recommendations for policy and/or practice supported by the reported data?	-	✓	✓	✓
Were the specific directives for new research appropriate?	✓	✓	✓	✓

^a^Both Hudon and Köberich did an extensive search for grey literature

### Overview of the validation studies

Sixty-eight validation studies were included across the four reviews. Hudon et al. [23] described one to three validation studies for each instrument included and was the only review to report specific information on the validation studies in addition to information on the instruments. Köberich and Farin [21] identified several validation studies for each instrument. Brouwers et al. [22] identified one validation study for each included instrument. Ekman et al. [24] describe one validation study for 13 instruments, and two validation studies for three other included instruments. Table 3 provides an overview of the validation studies [3, 27–91].

**Table 3:** Overview of validation studies (*n* = 68)

Instrument	Authors	Abbreviation	In which review	Year	Country	Type of study^b^	Own instrument^a^
4 Habits Coding Scheme	Frankel & Stein	4HCS	Ekman	2001	USA	Development & validation	Yes
4 Habits Coding Scheme	Krupat et al	4HCS	Ekman	2006	N/S	Validation	Yes
Modified version of The Roter Interaction Analysis System	Mjaaland et al	ARCS(RIAS)	Ekman	2009	Norway	Instrument modification	Yes
Biopsychosocial Tool	Margalit et al	BPS tool	Brouwers	2007	Israel	Development & validation	Yes
Consultation and Relational Empathy	Mercer et al	CARE	Hudon & Brouwers	2004	UK	Development & validation	Yes
Consultation and Relational Empathy	Mercer et al	CARE	Hudon	2005	UK	Validation	Yes
Consultation and Relational Empathy	Mercer et al	CARE	Hudon	2008	UK	Using the instrument	Yes
Client-Centred Care Questionnaire	de Witte et al	CCCQ	Koberich	2006	N/S	Development & validation	Yes
Little instrument	Little et al	Little instrument	Hudon & Brouwers	2001	UK	Development & validation	Yes
Little instrument	Little et al	Little instrument	Hudon	2001	UK	Development & validation	Yes
Little instrument	Smith & Orrell	Little instrument	Hudon	2007	UK	Using the instrument	No
Common Ground	Lang et al	CG	Brouwers	2004	USA	Development & validation	Yes
CARES Observational tool	Gaugler et al	COT	Ekman	2013	USA	Development & validation	Yes
Component of Primary Care Instrument	Flocke et al	CPCI	Hudon	1999	USA	Using the instrument	Yes
Component of Primary Care Instrument	Flocke et al	CPCI	Hudon	1998	USA	Using the instrument	Yes
Component of Primary Care Instrument	Flocke	CPCI	Hudon	1997	USA	Development & validation	Yes
Detail of Essential Elements and Participants in Shared Decision Making	Clayman et al	DEEP-SDM	Ekman	2012	USA	Development & validation	Yes
Davis Observation Code (Modified version)	Bertakis & Azari	DOC	Ekman	2011	USA	Instrument modification	Yes
General Practice Assessment Survey	Ramsay et al	GPAS	Hudon	2000	UK	Validation	Unclear
General Practice Assessment Survey	Jayasinghe et al	GPAS	Hudon	2008	Australia	Using the instrument	No
Henbest and Stewart instrument	Henbest & Stewart	Henbest and Stewart instrument	Ekman	1989	UK	Development & validation	Yes
Individualised Care Scale	Suhonen et al	ICS	Koberich	2005	N/S	Development & validation	Yes
Individualised Care Scale	Suhonen et al	ICS	Koberich	2000	Finland	Development & validation	Yes
Individualised Care Scale	Suhonen et al	ICS	Koberich	2012	N/S	Validation	Yes
Individualised Care Scale	Petroz et al	ICS	Koberich	2011	Canada	Validation	No
Individualised Care Scale	Acaroglu et al	ICS	Koberich	2011	Turkey	Translation and validation of instrument	Yes
Individualised Care Scale	Suhonen et al	ICS	Koberich	2010	Sweden	Translation and validation of instrument	Yes
Individualised Care Scale	Suhonen et al	ICS	Koberich	2000	Finland	Using the instrument	Yes
Informed Decision Making instrument	Braddock et al	IDM	Ekman	1997	USA	Development & validation	Yes
Instrument on Doctor-Patient Communicaton Skills	Campbell et al	IDPCS	Hudon	2007	Canada	Development & validation	Yes
Interpersonal Processes of Care	Stewart et al	IPC	Hudon	1999	USA	Development & validation	Yes
Interpersonal Processes of Care	Stewart et al	IPC	Hudon	2007	USA	Development & validation	Yes
Interpersonal Skills Rating Scale	Schnabl et al	IPS	Brouwers	1991	Canada	Development & validation	Yes
Medical Communication Competence Scale	Cegala et al	MCCS	Hudon	1998	USA	Development & validation	Yes
Measure of Patient-Centered Communication (Modified version)	Dong et al	MPCC	Ekman	2014	Australia	Instrument modification	No
Perceived Involvement in Care Scale (Modified)	Smith et al	M-PICS	Brouwers	2006	USA	Validation	No
Nonverbal Accommodation Analysis System	D’Agostino & Bylund	NAAS	Ekman	2011	USA	Development & validation	Yes
Nonverbal Accommodation Analysis System	D’Agostino & Bylund	NAAS	Ekman	2014	N/S	Using the instrument	Yes
North Worcestershire Vocational Training Scheme Patient Satisfaction Questionnaire	Jenkins & Thomas	NWVTS-PSC	Brouwers	1996	UK	Development & validation	Yes
Oncology Patients' Perception of the Quality of Nursing Care Scale	Radwin et al	OPPQNCS	Koberich	2003	N/S	Development & validation	Yes
Oncology Patients' Perception of the Quality of Nursing Care Scale	Suhonen et al	OPPQNCS	Koberich	2007	Finland	Validation	No
Oncology Patients' Perception of the Quality of Nursing Care Scale	Can et al	OPPQNCS	Koberich	2008	Turkey	Translation and validation of instrument	No
Oncology Patients' Perception of the Quality of Nursing Care Scale	Suhonen et al	OPPQNCS	Koberich	2007	Finland	Using the instrument	No
Observing patient involvement	Elwyn et al	OPTION	Ekman	2003	UK	Development & validation	Yes
Patient-centred Behaviour Coding Instrument	Zandbelt et al	PBCI	Ekman	2005	Netherlands	Development & validation	Yes
Primary Care Assessment Survey	Safran et al	PCAS	Hudon	2006	USA	Not a validation study	
Primary Care Assessment Survey (development of instrument not reported)	Safran et al	PCAS	Hudon	1998	USA	Validation	Unclear
Primary Care Assessment Survey	Duberstein et al	PCAS	Hudon	2007	USA	Using the instrument	No
Primary Care Assessment Tool—Adult	Shi et al	PCAT-A	Hudon	2001	USA	Validation	Unclear
Primary Care Assessment Tool—Adult	Haggerty et al	PCAT-A	Hudon	2008	Canada	Using the instrument	No
Patient-Centred Observation Form	Chesser et al	PCOF	Brouwers & Ekman	2013	USA	Validation	No
Patient-Centered Observation Form	Schirmer et al	PCOF	Ekman	2005	USA	Not a validation study	
Patient Feedback Questionnaire on Communication Skills (PFC is an adaptation of the PPPC)	Reinders et al	PFC	Brouwers	2009	Netherlands	Development & validation	Yes
Perceived Involvement in Care Scale	Lerman et al	PICS	Hudon	1995	USA	Development & validation	Yes
Perceived Involvement in Care Scale	Loh et al	PICS	Hudon	2007	USA	Using the instrument	No
Process of Interactional Sensitivity Coding in Healthcare	Sabee et al	PISCH	Ekman	2015	USA	Development & validation	Yes
Patient Perception of Patient-Centeredness	Mallinger et al	PPPC	Hudon	2005	USA	Not a validation study	
Patient Perception of Patient-Centeredness	Stewart et al	PPPC	Hudon	2000	Canada	Using the instrument	Yes
Patient Perception of Patient-Centeredness	Stewart et al	PPPC	Brouwers	2004	Canada	Unknown	Yes
Patient Perception of Quality	Haddad et al	PPQ	Hudon	2000	Canada	Development & validation	Yes
Patient Reactions Assessment	Galassi et al	PRA	Hudon	1992	USA	Development & validation	Yes
Quality of Communication	Engelberg et al	QoC	Brouwers	2006	USA	Validation	Yes
Questionnaire on the Quality of Physician–Patient Interaction	Bieber et al	QQPPI	Brouwers	2010	Germany	Development & validation	Yes
Relational Communication Scale for Observational measurement (Adapted versionod Burgoon and Hale)	Gallagher et al	RCS-O	Ekman	2001	USA	Instrument modification	No
Rochester Participatory Decision-Making Scale	Shields et al	RPAD	Ekman	2005	USA	Development & validation	Yes
Revised Patient-Centred Communication and Interpersonal Skills Scale (Revision of UCI scale into RUCIS)	Iramaneerat et al	RUCIS	Brouwers	2009	USA	Instrument modification	Yes
Smoliner Scale	Smoliner et al	Smoliner Scale	Koberich	2009	N/S	Development & validation	Yes
Sherbrooke Observation Scale of Patient-Centered Care	Paul-Savoie et al	SOS-PCC	Ekman	2015	Canada	Development & validation	Yes

*N/S* Not specified in review

^**a**^Own instrument = At least one of the validation study authors was involved in the development of the instrument

^b^Instrument modification: study describes a modification of the instrument (e.g., adaptation to a different setting); Using the instrument: the study uses the instrument as measurement in another study e.g., RCT

The validation studies were published between 1989 and 2015 inclusive. The majority of the studies were done in English speaking countries: 29 originated in the USA, 10 in the UK, 8 in Canada; 4 in Finland; 2 in Australia, the Netherlands, and Turkey; and 1 in Germany, Israel, Norway, and Sweden. The country of origin was not specified for the remaining 7 studies.

### Overview of the instruments

Forty-two instruments were included across the four reviews, with minimal overlap. The Patient-Centred Observation Form (PCOF) was included in two reviews [22, 24]. The original Perceived Involvement in Care Scale (PICS) is included by Hudon [23], while Brouwers [22]included the modified PICS (M-PICS). The Consultation and Relational Empathy instrument (CARE), and the Patient Perception of Patient Centeredness (PPPC) are included by both Hudon and Brouwers [22, 23]. Hudon [23] included what they referred to as the Consultation Care Measure (CCM), and Brouwers [22] included the same instrument, named differently as the Little instrument. Little et al. [34] do not name the instrument in their validation study, so we decided to refer to this instrument as the ‘Little Instrument’ in this review of reviews.

The four reviews reported varying types of information on the included instruments. All reported the year and country of development, the response scale, the number of subscales and items, and the intended rater of the instrument. Table 4 gives an overview of what information about the instrument is included in each review.

**Table 4:** Reported data on instruments included in each review

	Hudon 2011	Köberich 2015	Brouwers 2017	Ekman 2020
Origin (i.e., how was the instrument developed)	✓	✓	-	✓
Year of development	✓	✓	✓	✓
Country	✓	✓	✓	✓
Original language	-	✓	✓	-
Available in which languages	-	-	-	-
Conceptual framework or theoretical background	✓	✓	-	y/n only
Conceptual framework dimensions measured	✓	-	✓	✓
Development process details	-	✓	-	✓
Subscales/domains/categories	✓	✓	n only	✓
Items	✓	n only	n only	N/A
Response scale/scoring instructions	✓	✓	✓	✓
Rater	✓	✓	✓	✓
Instrument measurement aim	-	✓	✓	-
Format (e.g., checklist, coding system)	-	-	-	✓
Designed for educational purposes (y/n)	-	-	✓	-
Competency (= skill) measured	-	-	-	✓
COSMIN ratings	-	-	✓	-

As with the validation studies, the publication years of the instruments ranged from 1989 up to 2015. The majority of the instruments were developed in English speaking countries: 21 originated from the USA, 7 from the UK, 7 from Canada; 2 from the Netherlands; and 1 from Australia, Finland, Germany, Israel, and Norway. The country of origin was not specified in the review for the remaining 3 instruments. Table 5 summarises the information that is reported in the reviews.

**Table 5:** Overview of the instruments (*n* = 42)

Instrument	Review	Abbreviation	Developer	Year	Country	Subscales/categories N*	Items N	Response scale
BPS tool	Brouwers	BPS tool	Margalit et al	2007	Israel	3 subscales + 1 global item	9	0–100
Burgoon and Hale Relational Communication Scale for Observational Measurement (Adapted version)	Ekman	RCS-O	Gallagher et al	2001	USA	6	34	7-point
CARES Observational tool	Ekman	COT	Gaugler et al	2013	USA	0	16	0–1
Client-Centred Care Questionnaire	Koberich	CCCQ	de Witte et al	2006	N/S	0	15	5-point
Common Ground	Brouwers	CG	Lang et al	2004	USA	0	7	5-point
Components of Primary Care Instrument	Hudon	CPCI	Flocke et al	1997	USA	4	19	5-point
Consultation and Relational Empathy	Hudon & Brouwers	CARE	Mercer et al	2004	UK	0	10	5-point
Davis Observation Code (modified version)^a^	Ekman	DOC	Bertakis & Azari	2011	USA	6	20	N/A
Detail of Essential Elements and Participants in Shared Decision Making^b^	Ekman	DEEP-SDM	Clayman et al	2012	USA	10	-	9-point
Four Habits Coding Scheme	Ekman	4HCS	Frankel & Stein	2001	USA	4	23	5-point
General Practice Assessment Survey	Hudon	GPAS	Ramsay et al	2000	UK	9 subscales + 4 individual items	36	1–100
Henbest and Stewart instrument	Ekman	Henbest and Stewart instrument	Henbest & Stewart	1989	UK	0	15	4-point
Individualized Care Scale	Koberich	ICS	Suhonen et al	2000 (first version); 2010 (latest version)	Finland	2 parts 3 subscales each	34	5-point
Informed Decision Making instrument	Ekman	IDM	Braddock et al	1997	USA	6	N/A	0–1
Instrument on Doctor-Patient Communication Skills	Hudon	IDPCS	Campbell et al	2007	Canada	0	19	5-point
Interpersonal Processes of Care^c^	Hudon	IPC	Stewart et al	1999	USA	13	41	5-point
Interpersonal Skills Rating Scale	Brouwers	IPS	Schnabl et al	1991	Canada	0	13	7-point
Little instrument	Hudon & Brouwers	Little instrument	Little et al	2001	UK	5	21	4-point
Measure of Patient-Centered Communication (Modified version)	Ekman	MPCC	Dong et al	2014	Australia	2	15	6-point
Medical Communication Competence Scale	Hudon	MCCS	Cegala et al	1998	USA	4	24	7-point
Nonverbal Accommodation Analysis System^d^	Ekman	NAAS	D'Agostino & Bylund	2011	USA	10	N/A	N/A
North Worcestershire Vocational Training Scheme Patient Satisfaction Questionnaire	Brouwers	NWVTS-PSC	Jenkins & Thomas	1996	UK	0	11	5-point
Observing Patient Involvement	Ekman	OPTION	Elwyn et al	2003	UK	0	12	5-point
Oncology Patients’ Perceptions of the Quality of Nursing Care Scale^e^	Koberich	OPPQNCS	Radwin et al	2003	N/S	4	40	6-point
Patient Feedback Questionnaire on Communication Skills	Brouwers	PFC	Reinders et al	2009	Netherlands	0	16	4-point
Patient Perception of Patient Centeredness^f^	Hudon & Brouwers	PPPC	Stewart et al	2000/ 2004	Canada	4	14	4-point
Patient Perception of Quality	Hudon	PPQ	Haddad et al	2000	Canada	3	22	5-point
Patient Reactions Assessment	Hudon	PRA	Galassi et al	1992	USA	3	15	7-point
Patient-Centred Behaviour Coding instrument	Ekman	PBCI	Zandbelt et al	2005	Netherlands	2	N/A	N/A
Patient-Centred Observation Form	Brouwers & Ekman	PCOF	Chesser et al	2013	USA	13	N/A	3-point
Perceived Involvement in Care Scale	Hudon	PICS	Lerman et al	1995	USA	3	13	0–1
Perceived Involvement in Care Scale (Modified version)	Brouwers	M-PICS	Smith et al	2006	USA	4	20	5-point
Primary Care Assessment Survey	Hudon	PCAS	Saffran et al	1998	USA	11	51	1–100
Primary Care Assessment Tool (adult edition)	Hudon	PCAT-A	Shi et al	2001	USA	7	74	4-point
Process of Interactional Sensitivity Coding in Healthcare	Ekman	PISCH	Sabee et al	2015	USA	7	N/A	N/A
Quality of Communication	Brouwers	QoC	Engelberg et al	2006	USA	0	13	10-point
Questionnaire on the Quality of Physician–Patient Interaction	Brouwers	QQPPI	Bieber et al	2010	Germany	0	14	5-point
Revised Patient-Centred Communication and Interpersonal Skills Scale	Brouwers	RUCIS	Iramaneerat et al	2009	USA	0	13	4-point
Rochester Participatory Decision-Making Scale	Ekman	RPAD	Shields et al	2005	USA	0	9	3-point
Roter Interaction Analysis System (Modified version)^g^	Ekman	ARCS(RIAS)	Mjaaland & Finset	2009	Norway	14	N/A	N/A
Sherbrooke Observation Scale of Patient-Centered Care	Ekman	SOS-PCC	Paul-Savoie et al	2015	Canada	0	9	4-point
Smoliner scale	Koberich	Smoliner scale	Smoliner	2009	N/S	2	9	6-point

^*^The review by Ekman et al. only includes observation tools (checklists and coding schemes) which usually do not include subscales

^a^The DOC consists of 20 codes in 6 clusters

^b^The DEEP-SDM consists of 10 categories

^c^The IPC consists of 3 domains, 13 subscales, and 41 items. A shorter version with 7 subscales and 29 items is available

^d^The NAAS consists of 10 non-verbal behaviour categories

^e^A shorter 18 item version of the OPPQNCS is available

^f^A shorter 9 item version of the PPPC is available

^g^10 original RIAS categories, 4 ARCS categories

The measurement properties of instruments that were reported in the reviews varied considerably.. Table 6 shows which properties were reported in which review, and Table 7 is a literal presentation of all psychometric information reported in the four included reviews.

**Table 6:** Reported measurement properties of instruments

**Instrument**	**Abbreviation**	**Review**	**Reliability**			**Validity**											
			**Internal consistency**	**Test–retest**	**Inter-rater**	**Content**	**Structural**	**Construct**	**Convergent**	**Factor analysis**	**Known groups**	**Criterion**	**Cross-cultural**	**Face**	**Discriminant**	**Predictive**	**Concurrent**
BPS tool	BPS tool	Brouwers	✓			✓	✓										
Burgoon and Hale Relational Communication Scale for Observational Measurement (Adapted version)	RCS-O	Ekman	✓		✓												✓
CARES Observational tool	COT	Ekman			✓	✓								✓			
Client-Centred Care Questionnaire	CCCQ	Koberich	✓				✓	✓	✓	✓	✓						
Common Ground	CG	Brouwers	✓	✓			✓					✓					
Components of Primary Care Instrument	CPCI	Hudon	✓			✓										✓	
Consultation and Relational Empathy	CARE	Hudon & Brouwers	✓			✓						✓		✓		✓	✓
Consultation Care Measure	CCM	Hudon	✓														
Davis Observation Code (modified version)	DOC	Ekman															
Detail of Essential Elements and Participants in Shared Decision Making	DEEP-SDM	Ekman															
Four Habits Coding Scheme	4HCS	Ekman	✓		✓												✓
General Practice Assessment Survey	GPAS	Hudon	✓	✓											✓		
Henbest and Stewart instrument	Henbest and Stewart	Ekman		✓	✓												
Individualized Care Scale (4th version) – English version (Canada) (Petroz et al. 2011)	ICS	Koberich	✓				✓	✓		✓			✓				
Individualized Care Scale (4th version) – Finnish, Greek, Swedish and English version (Suhonen et al. 2010)	ICS	Koberich	✓				✓	✓	✓	✓							
Individualized Care Scale (4th version) – Turkish version (Acaroglu et al. 2011)	ICS	Koberich	✓				✓	✓		✓							
Informed Decision Making instrument	IDM	Ekman			✓												
Instrument on Doctor-Patient Communication Skills	IDPCS	Hudon	✓							✓				✓			
Interpersonal Processes of Care	IPC	Hudon	✓														
Interpersonal Skills Rating Scale	IPS	Brouwers	✓		✓		✓			✓							
Little instrument	Little instrument	Brouwers	✓				✓			✓							
Measure of Patient-Centered Communication (Modified version)	MPCC	Ekman	✓		✓	✓											✓
Medical Communication Competence Scale	MCCS	Hudon	✓														
Nonverbal Accommodation Analysis System	NAAS	Ekman		✓	✓	✓											✓
North Worcestershire Vocational Training Scheme Patient Satisfaction Questionnaire	NWVTS-PSC	Brouwers	✓			✓											
Observing Patient Involvement	OPTION	Ekman	✓	✓	✓	✓					✓						
Oncology Patients’ Perceptions of the Quality of Nursing Care Scale	OPPQNCS	Koberich	✓				✓	✓		✓							
Oncology Patients’ Perceptions of the Quality of Nursing Care Scale—Finnish version (Suhonen et al. 2007a,b)	OPPQNCS	Koberich	✓					✓	✓								
Oncology Patients’ Perceptions of the Quality of Nursing Care Scale—Short form – Turkish version (Can et al. 2008)	OPPQNCS	Koberich	✓														
Patient Feedback Questionnaire on Communication Skills	PFC	Brouwers	✓			✓	✓			✓							
Patient Perception of Patient Centeredness	PPPC	Brouwers	✓			✓						✓					
Patient Perception of Patient Centeredness (13 items)	PPPC	Hudon	✓														
Patient Perception of Quality	PPQ	Hudon	✓							✓					✓		
Patient Reactions Assessment	PRA	Hudon	✓							✓				✓	✓		
Patient-Centred behaviour coding instrument	PBCI	Ekman	✓		✓												✓
Patient-Centred Observation Form	PCOF	Brouwers & Ekman	✓	✓	✓												
Perceived Involvement in Care Scale	PICS	Hudon	✓							✓						✓	
Perceived Involvement in Care Scale (Modified version)	M-PICS	Brouwers	✓				✓						✓				
Primary Care Assessment Survey	PCAS	Hudon	✓														
Primary Care Assessment Tool	PCAT-A	Hudon	✓														
Process of Interactional Sensitivity Coding in Healthcare	PISCH	Ekman			✓									✓			
Quality of Communication	QoC	Brouwers	✓				✓										
Questionnaire on the Quality of Physician–Patient Interaction	QQPPI	Brouwers	✓	✓		✓	✓			✓							
Revised Patient-Centred Communication and Interpersonal Skills Scale	RUCIS	Brouwers															
Rochester Participatory Decision-Making Scale	RPAD	Ekman			✓												✓
Roter Interaction Analysis System (Modified version)	ARCS (RIAS)	Ekman			✓												✓
Sherbrooke Observation Scale of Patient-Centered Care	SOS-PCC	Ekman	✓		✓	✓											
Smoliner scale	Smoliner scale	Koberich	✓						✓		✓

**Table 7:** Data on measurement properties of instruments

Instrument	Abbreviation	Review	Reliability	Validity
BPS tool	BPS tool	Brouwers	Cronbach’s alpha = 0.90	Construct validity: interobserver variance between BPS-oriented physicians and biomed oriented physicians: range: 23.2–59.3 (*p* < 0.0001)
Burgoon and Hale Relational Communication Scale for Observational Measurement (Adapted version)	RCS-O	Ekman	Inter-rater-reliability (Cronbach ´s alpha): Immediacy/affection = 0.62; Similarity/depth = 0.51; Receptivity/trust = 0.72; Composure = 0.69; Formality = 0.02; Dominance = 0.34. Internal consistency (Cronbach ´s alpha): Immediacy/affection = 0.95; Similarity/depth = 0.84; Receptivity/trust = 0.94; Composure = 0.98; Formality = 0.92; Dominance = 0.60. Inter-rater-agreement (within group agreement coefficient): Immediacy/affection = 0.65; Similarity/depth = 0.72; Receptivity/trust = 0.86; Composure = 0.74; Formality = 0.58; Dominance = 0.78. N interactions: 20, N raters: 3	Concurrent validity: correlation with other measure (Interview Rating Scale): Immediacy/affection = 0.65; Similarity/depth = 0.50; Receptivity/trust = 0.76; Composure = 0.62; Formality = − 0.31; Dominance = − 0.26
CARES Observational tool	COT	Ekman	Inter-rater reliability: ICC = 0.77 N interactions: 5, N raters: 5	Face validity: PI with input from scientific advisors reviewed Content validity: panel of several interdisciplinary experts
Client-Centred Care Questionnaire	CCCQ	Koberich	Cronbach’s alpha: 0.94	Structural validity (EFA): One factor, Factor explains 58% of the variance. Hypothesis-testing Validity with known groups: Differences between clients of three organizations (*P* = 0.08). Differences between clients of two organizations (*P* = 0.049). Convergent validity: Correlation ‘client-centredness’ – ‘overall satisfaction’: *r* = 0.81
Common Ground	CG	Brouwers	Internal consistency: Pearson’s *r* = 0.91 and 0.95 (for raters 1 and 2, respectively) Intra-rater reliability: rater 1: Pearson’s *r* = 0.63 (overall case rating), 0.69 (overall case percentage score); rater 2: Pearson’s *r* = 0.87 (overall case rating), 0.78 (overall case percentage score) Inter-rater reliability: global rating overall case: Pearson’s *r* = 0.85, checklist percentage score overall case: *r* = 0.92	Construct validity: interobserver variance between year 3 students intensive and minimal curriculum + (*p* < 0.001); Concurrent validity (expert versus rater): Pearson’s *r* = 0.84 (overall performance). Criterion validity: Correlation of overall performance between expert and rater: 0.84
Components of Primary Care Instrument	CPCI	Hudon	Internal consistency: Cronbach’s α ranging from .68 to .79. Accumulated knowledge (7/7, α = .88), interpersonal communication (6/ 6, α = .75), advocacy (2/9, α = .88), family context(2/3, α = .82) and community context (2/2, α not available)	Content validity: A panel of experts evaluated the relevance of the items to the component they proposed to measure and assessed the items for clarity and conciseness. Predictive validity: CPCI was related with patient satisfaction. Interpersonal communication was associated with being more up to date on screening
Consultation and Relational Empathy	CARE	Hudon & Brouwers	Internal consistency: Cronbach’s alpha = 0.93	Face and content validity: Feedback from patients interviewed, the general practitioners, and the expert researchers led to a number of modifications. Based on earlier studies on theoretical concept of empathy and compared with BLESS. Patient and colleague GP interviews based on grounded theory approach, experts’ advice. Criterion validity: Pearson’s *r* = 0.85, *p* < 0.001 with RES; Pearson’s *r* = 0.84, *p* < 0.001 with BLESS. Predictive validity: General practitioner empathy is associated with patient enablement at contact consultation and a prospective relationship between patient enablement and changes in main complaint and well-being at 1 month. Concurrent validity: Strong correlations with the Reynolds Empathy Scale (RES) and the Barret-Lennard Empathy Subscale (BLESS)
Davis Observation Code (modified version)	DOC	Ekman	NR	NR
Detail of Essential Elements and Participants in Shared Decision Making	DEEP-SDM	Ekman	NR	NR
Four Habits Coding Scheme	4HCS	Ekman	Inter-rater reliability (Pearson correlation): Habit 1 = 0.70, Habit 2 = 0.80, Habit 3 = 0.71, Habit 4 = 0.69, Overall 0.72. Internal consistency reliability (Cronbach ´s alpha): Habit 1 = 0.71, Habit 2 = 0.51, Habit 3 = 0.81 and Habit 4 = 0.61. N interactions: 13, N raters: 2	Concurrent validity: correlation with other measure (RIAS). Habit 1 = − 0.07–0.28, Habit 2 = 0.08–0.37, Habit 3 = − 0.01–0.37, Habit 4 = 0.01–0.21
General Practice Assessment Survey	GPAS	Hudon	Internal consistency: All Cronbach’s alpha’s were above 0.70 (except for the trust scale = 0.69) Test–retest reliability: All 7 of the multi-item scales had test–retest correlations greater than the 0.70. access: 0.81; technical care: 0.89; communication: 0.85; inter-personal care: 0.83; trust: 0.83; knowledge of patient: 0.87; nursing care: 0.92). Communication (2/4, α = .90), interpersonal care (3/3, α = .93), trust (2/4, α = .69) and knowledge of patient (3/3, α = .91)	Discriminant validity: Respondents who were extremely satisfied scored significantly higher than those who were not
Henbest and Stewart instrument	Henbest & Stewart	Ekman	Inter-rater reliability: Spearman correlation = 0.91 Intra-rater reliability: Spearman correlation = 0.88 (after 2 weeks) and 0.63 (after 6 weeks) N interactions: 18 (inter-rater); 8 (intra-rater, 2 weeks); 12 (intra-rater, 12 weeks) N raters: 2	NR
Individualized Care Scale (4th version) – English version (Canada) (Petroz et al. 2011)	ICS	Koberich	ICS-A: 0.94 ICS-B: 0.94	Structural validity (EFA): Three factor for ICS-A and two factors for ICS-B. Factors accountable for 69.2% of the variance in ICS-A and 63.6% of the variance in ICS-B. Convergent validity: Schmidt Perception of Nursing Care Survey (SPNCS) was used (measuring patient satisfaction) Spearman’s Rho: SPNCS vs. ICS-A: 0.76 (95% CI: 0.72, 0.80); SPNCS vs. ICS-B: 0.80 (95% CI: 0.77, 0.83)
Individualized Care Scale (4th version) – Finnish, Greek, Swedish and English version (Suhonen et al. 2010)	ICS	Koberich	Finnish version: ICS-A: 0.92; ICS-B: 0.90; ClinB: 0.88; PersB: 0.78; DecB: 0.77 Greek version: ICS-A: 0.97; ClinA: 0.96; PersA: 0.90; DecA: 0.92 ICS-B: 0.97; ClinB: 0.96; PersB: 0.87; DecB: 0.89. Swedish version: ICS-A: 0.93; ClinA: 0.88; PersA: 0.84; DecA: 0.89 ICS-B: 0.92; ClinB: 0.88; PersB: 0.80; DecB: 0.84. UK version: ICS-A: 0.97; ClinA: 0.93; PersA: 0.86; DecA: 0.94 ICS-B: 0.95; ClinB: 0.94; PersB: 0.80; DecB: 0.85. USA version: ICS-A: 0.94; ClinA: 0.86; PersA: 0.88; DecA: 0.88 ICS-B: 0.93; ClinB: 0.90; PersB: 0.78; DecB: 0.78	Structural validity (EFA): Three factor for ICS-A and ICS-B, Factors accountable for n % of the variance 1) Finnish version: ICS-A: 61.9%; ICS-B: 58.2% 2) Greek version: ICS-A: 73.9%; ICS-B: 68.8% 3) Swedish version:ICS-A: 65.6%; ICS-B: 62.1% 4) UK version: ICS-A: 79.7%; ICS-B: 79.7% Cross-cultural validity (Rasch-Analysis): Measurement of invariance between the ICS versions of four countries: general congruence in item calibration patterns, but slight differences in the rank order
Individualized Care Scale (4th version) – Turkish version (Acaroglu et al. 2011)	ICS	Koberich	ICS-A: 0.92; ClinA: 0.86; PersA: 0.72; DecA: 0.83 ICS-B: 0.93; ClinB: 0.89; PersB: 0.80; DecB: 0.84	Structural validity (EFA): Three factor for ICS-A and ICS-B. Factors accountable for 65% of the variance in ICS-A and 62% of the variance in ICS-B
Informed Decision Making instrument	IDM	Ekman	Inter-rater reliability: Agreement = 77%. N interactions: 20, N raters: 3	NR
Instrument on Doctor-Patient Communication Skills	IDPCS	Hudon	Internal consistency: Cronbach’s α for the patient questionnaire was .69	Face validity: The initial instruments were administered to 4 specialists and 3 family doctors who, along with their patients, provided feedback. Factor analysis: For patients, 60% of the variance was explained by the first factor (process of communication) and 6% by the second (content of communication)
Interpersonal Processes of Care	IPC	Hudon	Internal consistency: Cronbach’s α coefficients ranging from .65 to .90. Hurried communication (5/5, α = .65), elicited concerns, responded (3/3, α = .80), explained results, medication (4/4, α = .81), patient-centered decision-making (3/3, α = .75) and compassionate, respectful (5/5, α = .71)	
Interpersonal Skills Rating Scale	IPS	Brouwers	Reliability coefficient: medical students 0.72 (range: 0.68–0.76), foreign medical graduates 0.83 (range: 0.68–0.93); internal medicine residents: 0.48 and 0.42	Construct validity: correlation other instrument (patient rating form) and IPS = 0.95 (*p* < 0.0001). Factor 1 (communication of information and patient participation) explained 62% of variance; factor 2 (empathy and jargon free communication) explained 10% of variance
Little instrument	CCM	Hudon	Communication and partnership (11/11, α = .96), personal relationship (3/3, α = .89), health promotion (2/2, α = .87), positive and clear approach to problem (3/3, α = .84) and interest in effect on life (2/2, α = .89)	Satisfaction was related to communication and partnership and positive approach. Enablement was more significantly related with interest in effect on life, health promotion, and positive approach. Positive approach was associated with reduced symptom burden at 1 month. Referrals were fewer if patients felt they had a personal relationship with their doctor
Little instrument	Little instrument	Hudon & Brouwers	Internal consistency: Cronbach’s alpha = 0.96 (communication and partnership), 0.89 (personal relationship), 0.87 (health promotion), 0.84 (positive and clear approach to the problem), 0.89 (interest in effect on life)	Four factors explained 93% of variance
Measure of Patient-Centered Communication (Modified version)	MPCC	Ekman	Inter-coder reliability: Krippendorff’s α for process categories = 0.86. Internal consistency reliability: Cronbach ‘s alpha = 0.48. N interactions: 56, N raters: NR	Content validity: Panel of radiation therapists and PCC researchers. Concurrent validity: Comparison with other measure (Patient-perceived patient centeredness), Pearson correlation = 0.01
Medical Communication Competence Scale	MCCS	Hudon	No subscale (24/40, α = .79 for information giving, α = .76 for information seeking, α = .85 for information verifying, and α = .92 for socioemotional communication	
Nonverbal Accommodation Analysis System	NAAS	Ekman	Inter-rater reliability (Pearson correlation): paraverbal = 0.81–0.96; nonverbal = 0.85–0.93. Intra-rater reliability (Pearson correlation): paraverbal = 0.82–1.0; non-verbal = 0.89–0.94. N interactions: 10, N raters: 2	Concurrent validity: correlation with other measure (MIPS): physician eye contact = 0.45; patient eye contact = 0.62
North Worcestershire Vocational Training Scheme Patient Satisfaction Questionnaire	NWVTS-PSC	Brouwers	Internal consistency: Cronbach’s alpha = 0.84	Content validity: Association with general satisfaction with the consultation Spearman’s r = 0.61 (exploring patient understanding), 0.54 (ease of problem sharing), 0.52 (sufficient time in consultation)
Observing Patient Involvement	OPTION	Ekman	Inter-rater reliability: ICC = 0.62; Cohen´s kappa = 0.71; Generalisability coefficient = 0.68. Intra-rater reliability: Generalisability coefficient = 0.66. Internal consistency reliability: Cronbach ´s alpha = 0.79. N interactions: 186, N raters: 2	Content validity: items formulated from existing literature. Known groups validity: scores influenced by patient age (negative); sex of clinician (positive in favour of female); qualification of clinician (positive), and clinical equipoise (positive)
Oncology Patients’ Perceptions of the Quality of Nursing Care Scale	OPPQNCS	Koberich	Internal consistency: Total scale: 0.99 (Short form: 0.97), Responsiveness: 0.99 (Short form: 0.95), Individualization: 0.97 (Short form: 0.93), Coordination: 0.87 (Short form: 0.87), Proficiency: 0.95 (Short form: 0.95)	Structural validity, EFA: Four factors: (1) Responsiveness, (2) Individualization, (3) Coordination, (4) Proficiency. Four factors explain 80.5% of the variance
Oncology Patients’ Perceptions of the Quality of Nursing Care Scale—Finnish version (Suhonen et al. 2007a,b)	OPPQNCS	Koberich	Internal consistency: Total scale: 0.94, Responsiveness: 0.91, Individualization: 0.87, Coordination: 0.85, Proficiency: 0.90	Convergent validity (Pearsons r): Correlation of OPPQNCS subscales assessing individualized care with ICS subscales assessing individualized care: *r* = 0.64/0.66. Correlation of OPPQNCS subscales assessing individualized care with Schmidt Perception of Nursing Care Survey subscales assessing individualized care: *r* = 0.67. Divergent validity (Pearsons r): Correlation of OPPQNCS subscales not assessing individualized care with ICS subscales assessing individualized care: *r* = 0.51–0.60. Correlation of OPPQNCS subscales not assessing individualized care with Schmidt Perception of Nursing Care Survey subscales assessing individualized care: *r* = 0.53–0.62
Oncology Patients’ Perceptions of the Quality of Nursing Care Scale—Short form – Turkish version (Can et al. 2008)	OPPQNCS	Koberich	Total scale: 0.91, Responsiveness: 0.74, Individualization: 0.79, Coordination: 0.66, Proficiency: 0.87	NA
Patient Feedback Questionnaire on Communication Skills	PFC	Brouwers	Internal consistency: Cronbach’s alpha = 0.89, item–total correlations ranged from 0.45 (question 11) to 0.67 (questions 9 and 13)	Construct validity: correlation original construct (translated PPPC) and new construct (PFC): 0.97. One factor explained 55.64% of variance
Patient Perception of Patient Centeredness	PPPC	Hudon	Alpha = .71	The PPPC showed significant correlations with better recovery from discomfort, alleviation of concerns, and better emotional health 2 months after the initial visit, and with use of fewer diagnostic tests and referrals. Patients’ perception of patient-centered behaviors was strongly associated with patients’ satisfaction with information
Patient Perception of Patient Centeredness (14 items)	PPPC	Brouwers	Internal consistency: Cronbach’s alpha = 0.71	Criterion validity: Pearson’s *r* = 0.16, *p* < 0.01 with MPCC
Patient Perception of Patient Centeredness (9 item)	PPPC	Brouwers	Internal consistency: Cronbach’s alpha = 0.80 (patient questionnaire), 0.79 (physician questionnaire)	
Patient Perception of Quality	PPQ	Hudon	Internal consistency: Cronbach’s α coefficients ranging from .83 to .94. Interpersonal aspects of care (5/5, α = .91) and technical aspects of care (5/12, α = .91)	Discriminant validity: Indices developed are potentially discriminating. Factor analysis: The 3 factors explained 60% of the total variance
Patient Reactions Assessment	PRA	Hudon	Overall Cronbach’s α of .91. Patient information index (2/5, α = .87), patient communication index (1/5, α = .91) and patient affective index (5/5, α = .90)	Face validity: An initial pool of 56 items was evaluated for face validity by 4 oncologist nurses and 13 counselling students. Discriminant validity: PRA was able to differentiate a group of providers who were perceived by counselling professionals as having more effective relationships with patients from a group who were perceived as having less effective patient relationship. Factor analysis: The 3-factor oblique model seemed to provide the best fit to the data
Patient-Centred Behaviour Coding instrument	PBCI	Ekman	Inter-rater reliability (ICC); Relative agreement: facilitating = 0.93, inhibiting = 0.53; Absolute agreement: facilitating = 0.92, inhibiting = 0.53. Internal consistency reliability (Cronbach ´s alpha): facilitating = 0.64, inhibiting = 0.50. N interactions: 323, N raters: 4	Concurrent validity: Correlation with other measure (Euro communication): facilitating (*r* = 0.28 and inhibiting (*r* = − 0.29)
Patient-Centred Observation Form	PCOF	Brouwers & Ekman	Overall inter-rater reliability Cronbach’s alpha = 0.67. N interactions: 13, N raters: 4. clinician’s inter-rater reliability: 0.45; social scientist’s inter-rater reliability: 0.62	NR
Perceived Involvement in Care Scale	PICS	Hudon	Internal consistency: Overall Cronbach’s α of .73. Doctor facilitation (5/5, α = .60-.73)	Predictive validity: Doctor facilitation and patient decision making were related with patient satisfaction with care. Doctor facilitation and information exchange was related with patients’ control over illness, and expectations for improvement in functioning. Doctor facilitation scale was related with patient participation Factor analysis: 3 relatively independent factors
Perceived Involvement in Care Scale (Modified version)	M-PICS	Brouwers	Internal consistency: Cronbach’s alpha = 0.87 (ranges: 0.79–0.89 (English), 0.76–0.86 (Spanish))	Convergent validity: Pearson’s *r* = -0.302, *p* < 0.01 (patient decision making and age); *r* = -0.314, *p* < 0.01 (facilitation and Latina status); *r* = 0.363, *p* < 0.001 (health care provider info and Latina); *r* = 0.0376, *p* < 0.001 (health care provider info and SES). Factor 1 (health care provider info) explained 32.01%, factor 2 (patient info) explained 16.42%, factor 3 (patient decision making) explained 9.45%, factor 4 (health care provider facilitation) explained 7.32%; total variance explained: 65.2%
Primary Care Assessment Survey	PCAS	Hudon	Internal consistency: Cronbach’s α ranging from .81 to .95. Contextual knowledge of patient (5/5, α = .92), communication (6/6, α = .95), interpersonal treatment (4/5, α = .95) and trust (5/8, α = .86)	
Primary Care Assessment Tool (adult edition)	PCAT-A	Hudon	Internal consistency: Cronbach’s α ranging from .64 to .95. Ongoing care (12/20, α = .92)	Content validity: 9 expert were asked to rate the appropriateness and representativeness of the primary care domain items. Factor analysis: 7 factors explained 88% of the total variance
Process of Interactional Sensitivity Coding in Healthcare	PISCH	Ekman	Inter-rater reliability: Cohen ´s kappa = 0.46–0.72; Scotts ´s pi = 0.44–0.72. N interactions: 50, N raters: NR	Face validity: review by panel of experts
Quality of Communication	QoC	Brouwers	Internal consistency: Cronbach’s alpha = 0.50	Convergent validity: Spearman’s *r* = 0.738 with overall quality of doctor’s communication and *r* = 0.432 with overall quality of discussions of end-of-life care (both p ≤ 0.000)
Questionnaire on the Quality of Physician–Patient Interaction	QQPPI	Brouwers	Internal consistency: Cronbach’s alpha = 0.95, Test–retest reliability: Pearson’s r = 0.59	Content: + + (adequate). Structural: PICS-A and SWD: *r* = 0.64 and 0.59 (*n* = 147), QHC and PICS-B: *r* = 0.54 and 0.52 (*n* = 147), PSHC: *r* = 0.38 (*n* = 147). One factor explained 60.11% of variance
Revised Patient-Centred Communication and Interpersonal Skills Scale	RUCIS	Brouwers	NA (tested using IRT—Rasch model)	NA (tested using IRT—Rasch model)
Rochester Participatory Decision-Making Scale	RPAD	Ekman	Inter-rater reliability: ICC = 0.72. N interactions: 193, N raters: NR	Concurrent validity: correlation with other measure (MPCC, dimension finding common ground) *r* = 0.19. Correlation with standardized patient perceptions (*r* = 0.32–0.36) and patient survey measures (*r* = 0.06–0.07)
Roter Interaction Analysis System (Modified version)	ARCS(RIAS)	Ekman	Inter-rater reliability (Cohen ´s kappa): 0.52. N interactions: 145, N raters: 5	Concurrent validity: correlation with other measure (RIAS). No misclassification between RIAS codes and ARCS codes
Sherbrooke Observation Scale of Patient-Centered Care	SOS-PCC	Ekman	Inter-rater reliability: ICC = 0.93. Internal consistency reliability: Cronbach ´s alpha = 0.88. N interactions: 42, N raters: 3	Content validity: 7 interdisciplinary experts in the health care field
Smoliner scale	Smoliner scale	Koberich	Total scale: n/a. Preferences: 0.84, Experiences: 0.86	Hypothesis-testing. Validity with known-groups: Group 1: experience with decision making = preference of decision-making; Group 2: experience with decision making ≠ preference of decision-making. Groups differ in overall satisfaction with decision-making (*P* < 0.001). Convergent validity: Correlation ‘experiences’ – ‘patient satisfaction with information process’: *r* = 0.673. Correlation ‘preferences’ – ‘patient satisfaction with information process’: *r* = 0.358

## Discussion

This review of reviews sought to summarise the range of validated instruments available for measuring practitioners’ person-centred consultation skills, including the strength of the validation evidence for each instrument, and to appraise the systematic reviews examining the validation studies. The reviews varied in quality, and our JBI quality assessment showed only one review which fulfilled all assessment criteria except for the assessment of publication bias [22]. In addition, only one review described several validation studies per instrument, including modifications and translations [21]. We found that the four included systematic reviews used very different inclusion criteria, leading to little overlap in included validation studies and instruments between them. This was because the reviews also differed in aims, appraisal tools used, and conceptual framework used, which limited the consistency of reported information across studies and instruments. These features underline the value of the present study, which in bringing together these literatures offers a guide to a wider set of instruments of interest to researchers than has previously been available. This diversity also underlines a key limitation of this review of reviews, as the included reviews themselves may complicate attention to the primary literature unhelpfully.

We make no claim that the list of instruments reported in this review of reviews is exhaustive. Our search was undertaken in September 2020 and although we have checked for citations of the included reviews and the primary studies, we may have missed later published reviews and instruments. There are many more instruments available, varying in aims, objectives, and conceptualisations of person-centredness. In addition, there may be other validation studies available on the instruments the reviews did not include, or which were published after the reviews, and the study findings suggest it is indeed likely that new instruments will have been published. We searched for all reviews meeting our selection criteria and acknowledge the perennial possibility that we may have missed eligible reviews, as well as being clear that there exist other validation studies and instruments that our study was not designed to include. We used an extensive list of keywords for our search, based on published reviews of person-centredness, but as the concept is so scattered, we may have left out search terms that could have led us to other reviews that could have been included. This we regard as a real risk and suggest careful extension of search strategy development in future studies. Procedural issues, particularly reliance on sole author for screening and data extraction, albeit with checks, should be borne in mind as review limitations.

There are many instruments available which measure person-centred skills in healthcare practitioners. The reviews point out that the instruments measured person-centredness in various dimensions, emphasising different aspects of the basic concept of person-centredness. This indicates the lack of agreement on what could be considered defining, central or important characteristics, so there are construct validity issues to be considered carefully. Person-centred care is an umbrella term used for many different conceptualisations in many different contexts [1, 4]. Separating consideration of what constitutes person centred care from person centred consultation skills is necessary, as the latter construct is merely one element of the former. Often teaching materials and guidelines on person centredness are not very clear on what person-centred behaviour and communication actually entails, and what skills and behaviours health care professionals are supposed to learn to make their practice person-centred. For example, Kitson and colleagues [13] reported that health policy stakeholders and nurses perceive patient-centred care more broadly than medical professionals. Medical professionals tend to focus on the doctor-patient relationship and the decision-making process, while in the nursing literature there is also a focus on patients’ beliefs and values [13]. Measurement instruments can help us operationalise person-centredness and can help practitioners understand what exactly it is that they are supposed to be doing. Developing the science of measurement in this area may also assist resolution of the construct validity issues by making clear what can be validly measured and what cannot.

Three of the four reviews [20, 21, 23] concluded that psychometric evidence is lacking for nearly all of the instruments. This finding may seem unsurprising in light of the foregoing discussion of construct validity. Brouwers [22] used the COSMIN rating scale [26] and found only one instrument rated as ‘excellent’ on all aspects of validity studied (internal consistency, content, and structural validity), but its reliability had not been studied. Köberich [21] specifically mentions test–retest reliability as a neglected domain and adds that all instruments lack evidence of adequate convergent, discriminant, and structural validity testing. Köberich and Farin, Brouwers, and Ekman [21, 22, 24] also highlight the need for further research on validity and reliability of existing instruments in their discussion and conclusion sections. In other reviews, De Silva [92], Gärtner et al. [93] and Louw et al. [94] attribute the lack of good evidence on the measurement qualities of instruments both to a failure to study their measurement properties and to the overall poor methodological quality of validation studies. Many tools are developed but few are studied sufficiently in terms of their psychometric properties and usefulness for research on and teaching of person-centredness. Often, a tool is “developed, evaluated, and then abandoned” [92].

Researchers and research users may seek instruments of these kinds for many different purposes. Using the most relevant and promising instruments that have already been developed and tested, in however a limited fashion, and rigorously studying and reporting on their psychometric properties, will be useful in building the science of measuring person-centred consultation skills. It may also be useful to develop item banking approaches that combine instruments. Researchers or educators intending to choose an instrument for their purposes also need to know several things to decide whether an instrument is relevant and suitable for their specific needs. For future primary studies and systematic reviews, we suggest paying heed to, and indeed rectifying, the limitations of existing studies identified here and elsewhere. In addition, both Hudon and Ekman [23, 24] found that paradoxically, there is very limited evidence of patients taking part in the evaluation process. This has also been reported in a systematic review by Ree et al. [95] who looked specifically at patient involvement in person centeredness instruments for health professionals. This is painfully ironic. There is thus a further major lesson to be drawn from this study; that in developing the science of measurement of person-centred skills, new forms of partnership need to be formed between researchers and patients.

## Conclusion

There are many instruments available which measure person-centred skills in healthcare practitioners and the most relevant and promising instruments that have already been developed, or items within them, should be further studied rigorously. Validation study of existing material is needed, not the development of new measures. New forms of partnership are needed between researchers and patients to accelerate the pace at which further work will be successful.

## Supplementary Information

**Additional file 1.** Search string for Embase, PsycInfo, MEDLINE. Search string for CINAHL.

## Data Availability

The datasets used and/or analysed during the current study are available from the corresponding author on reasonable request, as are template data collection forms; data extracted from included studies; data used for all analyses; analytic code; any other materials used in the review not provided here.

## Abbreviations

COSMIN: COnsensus-based Standards for the selection of health Measurement Instruments
STARD: Modified Version of Standards for Reporting of Diagnostic Accuracy
