# Supplementary material for: Measurement of person-centred consultation skills among healthcare practitioners: a systematic review of reviews of validation studies
Source: BMC Med Educ. 2023 Apr 5;23:211. doi: 10.1186/s12909-023-04184-6 (PMC10074817; doi:10.1186/s12909-023-04184-6)
Supplement: Supplementary file 1 — Additional file 1. Search string for Embase, PsycInfo, MEDLINE. Search string for CINAHL. [file 12909_2023_4184_MOESM1_ESM.docx]

**Search string for Embase, PsycInfo, MEDLINE**

|  | Person-cent* or patient-cent* or personcent* or patientcent* or person-orient* or person-focus* or person-participation or person-empowerment or person-involvement or patient-orient* or patient-focus* or patient-participation or patient-empowerment or patient-involvement or "person orient*" or "person focus*" or "person participation" or "person empowerment" or "person involvement" or "patient orient*" or "patient focus*" or "patient participation" or "patient empowerment" or "patient involvement" |
| --- | --- |
| OR | ((clinician-patient or physician-patient or professional-patient or provider-patient or practitioner-patient or pharmacist-patient or doctor-patient or nurse-patient) adj2 (communication* or consultation* or practice* or relation* or interaction* or rapport)) |
|  | 1 OR 2 |
| AND | psychometr* or clinimetr* or clinometr* or "outcome assessment" or "outcome measure" or "observer variation" or reproducib* or reliab* or unreliab* or valid* or coefficient or homogeneity or homogeneous or "internal consistency" or agreement or precision or imprecision or "precise values" or test-retest or stability or interrater or inter-rater or intrarater or intra-rater or intertester or inter-tester or intratester or intra-tester or interobserver or inter-observer or intraobserver or intra-observer or intertechnician or inter-technician or intratechnician or intra-technician or interexaminer or inter-examiner or intraexaminer or intra-examiner or interassay or inter-assay or inter-assay or intraassay or intra-assay or interindividual or inter-individual or intraindividual or intra-individual or interparticipant or inter-participant or intraparticipant or intra-participant or kappa or kappa's or kappas or repeatab* or generaliza* or generalisa* or concordance or discriminative or "known group" or "factor analys*" or dimension* or "subscale* item discriminant" or "interscale correlation*" or error* or "individual variability" or "standard error of measurement" or sensitiv* or responsive* or "meaningful change" or "ceiling effect" or "floor effect" or "item response model" or IRT or rasch or "differential item functioning" or DIF or "computer adaptive testing" or "item bank" or "cross cultural equivalence" or "cronbach* alpha*" or "replicab* measure*" or "replicab* finding*" or "replicab* result*" or "replicab* test* or repeated measure* or repeated finding* or repeated result* or repeated test* or item correlation* or item selection* or item reduction* or test retest or intraclass correlation* or multitrait scaling analys* or uncertainty measure*" or "variability analys* or variability value* or minimal* important change or minimal* important difference* or minimal* significant change or minimal* significant difference or minimal* detectable change or minimal* detectable difference or clinical* important change or clinical* important difference or clinical* significant change or clinical* significant difference or clinical* detectable change or clinical* detectable difference or small* real change or small* real difference" or "small* detectable change" or "small* detectable difference" |
| AND | ((systematic* or scoping) adj2 review*).ti, ab |

**Search string for CINAHL**

|  | Person-cent* or patient-cent* or personcent* or patientcent* or person-orient* or person-focus* or person-participation or person-empowerment or person-involvement or patient-orient* or patient-focus* or patient-participation or patient-empowerment or patient-involvement or "person orient*" or "person focus*" or "person participation" or "person empowerment" or "person involvement" or "patient orient*" or "patient focus*" or "patient participation" or "patient empowerment" or "patient involvement" |
| --- | --- |
| OR | ((clinician-patient or physician-patient or professional-patient or provider-patient or practitioner-patient or pharmacist-patient or doctor-patient or nurse-patient) N2 (communication* or consultation* or practice* or relation* or interaction* or rapport)) |
|  | 1 OR 2 |
| AND | psychometr* or clinimetr* or clinometr* or "outcome assessment" or "outcome measure" or "observer variation" or reproducib* or reliab* or unreliab* or valid* or coefficient or homogeneity or homogeneous or "internal consistency" or agreement or precision or imprecision or "precise values" or test-retest or stability or interrater or inter-rater or intrarater or intra-rater or intertester or inter-tester or intratester or intra-tester or interobserver or inter-observer or intraobserver or intra-observer or intertechnician or inter-technician or intratechnician or intra-technician or interexaminer or inter-examiner or intraexaminer or intra-examiner or interassay or inter-assay or inter-assay or intraassay or intra-assay or interindividual or inter-individual or intraindividual or intra-individual or interparticipant or inter-participant or intraparticipant or intra-participant or kappa or kappa's or kappas or repeatab* or generaliza* or generalisa* or concordance or discriminative or "known group" or "factor analys*" or dimension* or "subscale* item discriminant" or "interscale correlation*" or error* or "individual variability" or "standard error of measurement" or sensitiv* or responsive* or "meaningful change" or "ceiling effect" or "floor effect" or "item response model" or IRT or rasch or "differential item functioning" or DIF or "computer adaptive testing" or "item bank" or "cross cultural equivalence" or "cronbach* alpha*" or "replicab* measure*" or "replicab* finding*" or "replicab* result*" or "replicab* test* or repeated measure* or repeated finding* or repeated result* or repeated test* or item correlation* or item selection* or item reduction* or test retest or intraclass correlation* or multitrait scaling analys* or uncertainty measure*" or "variability analys* or variability value* or minimal* important change or minimal* important difference* or minimal* significant change or minimal* significant difference or minimal* detectable change or minimal* detectable difference or clinical* important change or clinical* important difference or clinical* significant change or clinical* significant difference or clinical* detectable change or clinical* detectable difference or small* real change or small* real difference" or "small* detectable change" or "small* detectable difference" |
| AND | TI ((systematic* or scoping) N2 review*) OR AB ((systematic* or scoping) N2 review*) |
